# Context-Dependent Role of Mitochondrial Fusion-Fission in Clonal Expansion of mtDNA Mutations

**DOI:** 10.1371/journal.pcbi.1004183

**Published:** 2015-05-21

**Authors:** Zhi Yang Tam, Jan Gruber, Barry Halliwell, Rudiyanto Gunawan

**Affiliations:** 1 Lee Kong Chian School of Medicine, Nanyang Technological University, Singapore; 2 Department of Biochemistry, Centre for Life Sciences, National University of Singapore, Singapore; 3 Yale-NUS College, Science Division, National University of Singapore, Singapore; 4 Institute for Chemical and Bioengineering, ETH Zurich, Zurich, Switzerland; 5 Swiss Institute of Bioinformatics, Lausanne, Switzerland; Rutgers University, UNITED STATES

## Abstract

The accumulation of mutant mitochondrial DNA (mtDNA) molecules in aged cells has been associated with mitochondrial dysfunction, age-related diseases and the ageing process itself. This accumulation has been shown to often occur clonally, where mutant mtDNA grow in number and overpopulate the wild-type mtDNA. However, the cell possesses quality control (QC) mechanisms that maintain mitochondrial function, in which dysfunctional mitochondria are isolated and removed by selective fusion and mitochondrial autophagy (mitophagy), respectively. The aim of this study is to elucidate the circumstances related to mitochondrial QC that allow the expansion of mutant mtDNA molecules. For the purpose of the study, we have developed a mathematical model of mitochondrial QC process by extending our previous validated model of mitochondrial turnover and fusion-fission. A global sensitivity analysis of the model suggested that the selectivity of mitophagy and fusion is the most critical QC parameter for clearing *de novo* mutant mtDNA molecules. We further simulated several scenarios involving perturbations of key QC parameters to gain a better understanding of their dynamic and synergistic interactions. Our model simulations showed that a higher frequency of mitochondrial fusion-fission can provide a faster clearance of mutant mtDNA, but only when mutant–rich mitochondria that are transiently created are efficiently prevented from re-fusing with other mitochondria and selectively removed. Otherwise, faster fusion-fission quickens the accumulation of mutant mtDNA. Finally, we used the insights gained from model simulations and analysis to propose a possible circumstance involving deterioration of mitochondrial QC that permits mutant mtDNA to expand with age.

## Author Summary

Mitochondria are responsible for most energy generation in human and animal cells. Loss or pathological alteration of mitochondrial function is a hallmark of many age-related diseases. Mitochondrial dysfunction may be a central and conserved feature of the ageing process. As part of quality control (QC), mitochondria are continually replicated and degraded. Furthermore, two mitochondria can fuse to form a single mitochondrion, and a mitochondrion can divide (fission) into two separate organelles. Despite this QC, mutant mitochondrial DNA (mtDNA) molecules have been observed to accumulate in cells with age which may lead to mitochondrial dysfunction. In this study, we created a detailed mathematical model of mitochondrial QC and performed model simulations to investigate circumstances allowing or preventing the accumulation of mutant mtDNA. We found that more frequent fusion-fission could quicken mutant mtDNA clearance, but only when mitochondria harboring a high fraction of mutant molecules were strongly prevented from fusing with other mitochondria and selectively degraded. Otherwise, faster fusion-fission would actually enhance the accumulation of mutant mtDNA. Our results suggested that the expansion of mutant mtDNA likely involves a decline in the selectivity of mitochondrial degradation and fusion. This insight might open new avenues for experiment and possible development of future therapies.

## Introduction

Mitochondria are multi-functional organelles of eukaryotic cells. While their main function is to generate ATP through oxidative phosphorylation (OXPHOS), mitochondria also participate in fatty acid oxidation, apoptosis, the cell cycle, and cell signaling [1]. Because of the importance of mitochondria, loss of mitochondrial function is detrimental to the organismal well-being. Mitochondrial dysfunction has been associated with a wide range of diseases, such as cancer, diabetes, presbycusis, sarcopenia, and neurodegenerative diseases [2, 3].

Mitochondria possess their own genome, mitochondrial DNA (mtDNA), encoding proteins that are involved in OXPHOS [4]. A single eukaryotic cell can harbor hundreds to thousands of mtDNA molecules [5], a number that is tightly regulated [6] and dependent on the cell type and metabolic requirement of the cell [7]. Mutations in mtDNA, including point mutations, rearrangements and deletions, can cause defects in the OXPHOS process. Wild-type (WT) and mutant mtDNA can coexist in a cell, a condition known as heteroplasmy, and each cell in a tissue may not have the same composition of mtDNA. Mutant mtDNA molecules have also been shown to accumulate with age in a variety of tissues and organisms [8], possibly contributing to the general age-related decline in mitochondrial function observed in almost all tissues [9]. However, because of complementation by WT mtDNA, mutant mtDNA molecules need to exceed a threshold between 60–90% before any phenotypic defect manifests [10]. It is therefore important to understand the processes involved in the quality control of mtDNA integrity.

Mitochondrial turnover and fusion-fission are key cellular mechanisms involved in the maintenance of mtDNA integrity and mitochondrial function [11]. Mitochondria are continuously turned over by the complementary processes of mitochondrial biogenesis (mitogenesis) and mitochondrial autophagy (mitophagy). Mitogenesis is regulated by a nuclear encoded protein family, the peroxisome proliferator-activated receptor gamma coactivator (PGC), e.g. PGC-1α and PGC-1β [12]. The rate of mitogenesis is tightly regulated in response to external stimuli and cellular stress through mitochondrial retrograde signalling [13]. Meanwhile, mitophagy is a selective process, in which mitochondria with lowered mitochondrial membrane potential, an indication of OXPHOS impairment, are preferentially removed [14, 15]. Together, mitogenesis and mitophagy form the backbone of the mitochondrial quality control process.

Perturbations of mitochondrial turnover have been shown to affect mitochondrial function and mtDNA. For example, upregulation of PGC-1α has been shown to lead to higher ATP production in wild-type mice, and delayed onset of myopathy and lifespan extension in a mouse model of mitochondrial myopathy [16]. Similarly, overexpression of PGC-1α in the mtDNA mutator mouse, a transgenic mouse carrying proof-reading deficient mtDNA polymerase *γ*, improved mitochondrial function despite a slightly higher mtDNA point mutation burden than wild-type mouse [17]. On the other hand, the number of mitochondria with lowered membrane potential increased upon downregulation of autophagy [18]. Meanwhile, rapamycin treatment has been shown to improve mitochondrial function possibly in part through an upregulation of autophagy [19] and increased autophagosomal degradation of mitochondria harboring mtDNA with mutations with a severe OXPHOS deficiency [20].

Mitochondrial fusion and fission are the processes by which two mitochondria combine to form a single organelle and a mitochondrion divides to form two mitochondria, respectively [21]. Together the mitochondrial fusion-fission enables the exchange of mitochondrial components among mitochondria organelles in a cell, including solutes, metabolites and proteins [22, 23], and mtDNA [24]. Such exchange provides a buffer against the impact of mtDNA mutations because protein products of WT mtDNA can complement proteins that are missing or defective due to mutations [25]. Disruptions to the machinery of mitochondrial fusion-fission have been shown to affect mitochondria-related function, such as the electron transport chain [26] and apoptosis [27], and have been associated with neurodegenerative and metabolic diseases [28]. A few experimental studies have also shown that mitochondrial fusion-fission can play a pivotal role in the maintenance of mtDNA integrity. For example, in C. *elegans*, the removal of helix-distorting mtDNA lesions induced by ultraviolet C (UVC) light depends on mitochondrial fusion-fission genes (*fzo-1*, *eat-3*, and *drp-1*) [29]. In addition, a severe depletion of either fusion or fission proteins has also been observed to cause a rapid accumulation of deleterious mtDNA mutations and loss of mitochondrial functions in mice and cell culture studies [30, 31].

A synergistic interaction exists between mitochondrial fusion and mitophagy in the mitochondrial quality control (QC) [15, 29, 32]. Specifically, depolarized mitochondria with lowered membrane potential have been shown to be less likely to undergo fusion than polarized mitochondria [15]. This selectivity involves the recruitment of Parkin, a cytosolic ubiquitin ligase, to depolarized mitochondria by mitochondrial outer membrane fusion proteins, mitofusins (Mfn1-2). The binding of Parkin to mitofusins depends on PTEN-induced putative kinase 1 (PINK1) protein. In addition, PINK1 phosphorylates mitofusins, promoting their Parkin-mediated ubiquitination and degradation [33–35]. The selectivity in fusion then leads to the segregation of depolarized mitochondria from the mitochondrial population in the cell. Finally, Parkin localization to damaged mitochondria promotes the targeted removal of these mitochondria through (selective) mitophagy [36]. Despite the presence of mitochondrial QC processes, mutant mtDNA molecules often accumulate with age leading to the loss of mitochondrial respiration. The circumstance permitting the expansion of mutant mtDNA is not precisely known, but important in formulating an intervention to the decline in mitochondrial function due to mtDNA mutation accumulation.

The focus of this study is to investigate the possible scenario, specifically involving deteriorations in the mitochondrial QC mechanism, that allows the expansion of mtDNA mutations. For this purpose, we would need a comprehensive understanding of how each mitochondrial QC process affects the removal and accumulation of mutant mtDNA. However, a systematic study of the processes involved in mitochondrial QC and their interactions is experimentally challenging. Here, the use of a computational approach through the creation of mathematical models and the simulations and analysis of such models becomes indispensable as an avenue to test hypotheses. For example, we and others have previously simulated mtDNA turnover and the occurrence of clonal expansion of mutant mtDNA due to random segregation of mtDNA populations [37, 38]. In addition, mathematical models of the fusion-fission process have been previously used to study how the health of mitochondrial populations varies with the rates of mitochondrial turnover and fusion-fission [39, 40]. In these models, mitochondrial population in a cell has been assumed to be well-mixed. More recently, in separate publications, we and Patel et al. have modeled the fusion-fission among spatially distributed mitochondria organelles in a cell [41, 42]. We have shown that the ability of mitochondrial retrograde signaling to slow down clonal expansion of mtDNA mutations requires an effective fusion-fission process.

In this study, we created a mathematical model of mitochondrial QC mechanism, by extending our previous model of mitochondrial fusion-fission process [42]. We employed a global sensitivity analysis to map out the dependence of mtDNA mutation burden on individual parameters of the mitochondrial QC. Using the corresponding sensitivity coefficients, we could determine the most important process(es) which, if perturbed, would significantly affect the ability of the QC to clear mtDNA mutations. Based on the results of model analysis and simulations, we finally propose a scenario that explains the accumulation of mtDNA mutations with age and we suggest potential intervention strategies to counter this accumulation.

## Results

**Fig 1:**
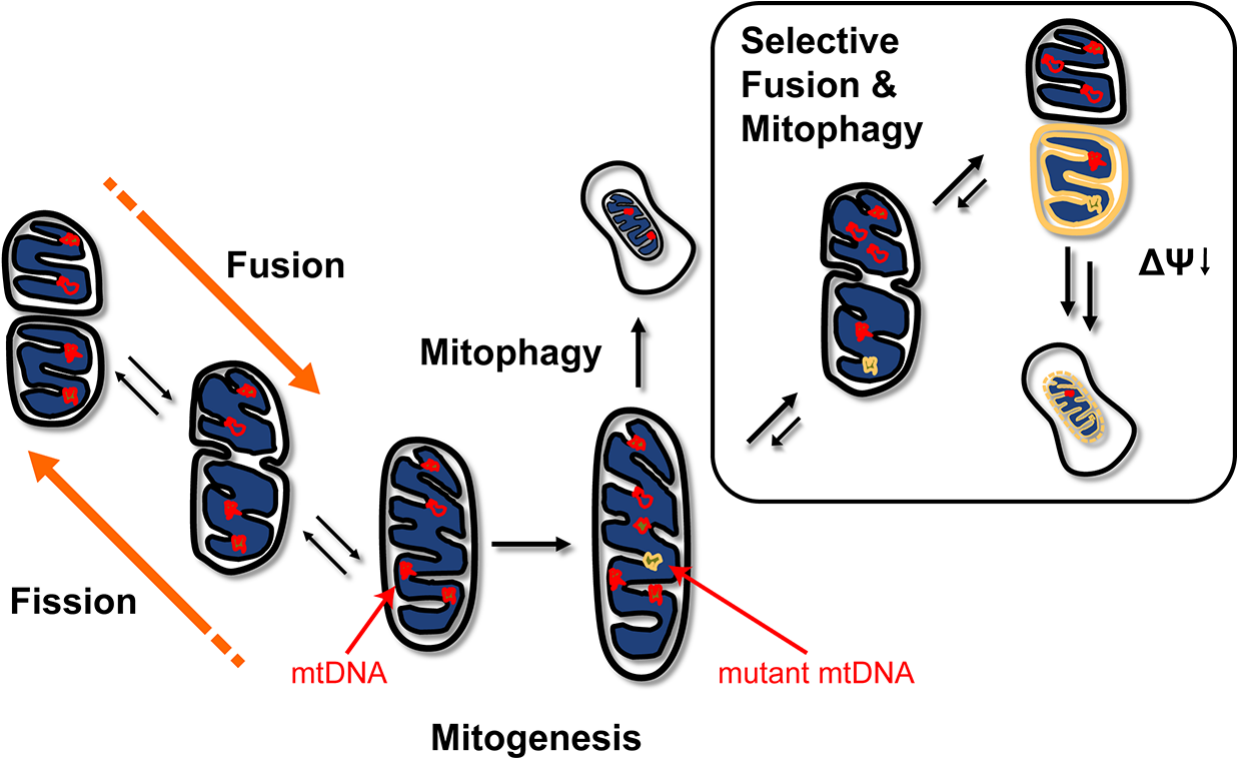
Mitochondrial quality control processes. The model accounts for the processes of mitochondrial turnover (mitogenesis and mitophagy) and mitochondrial fusion-fission. The box highlights the selectivity of mitochondrial fusion and mitophagy. Mitochondria with a high fraction of mutant mtDNA and consequently lowered membrane potential (Δψ) are less likely to fuse with other mitochondria and preferentially removed by mitophagy.

**Fig 2:**
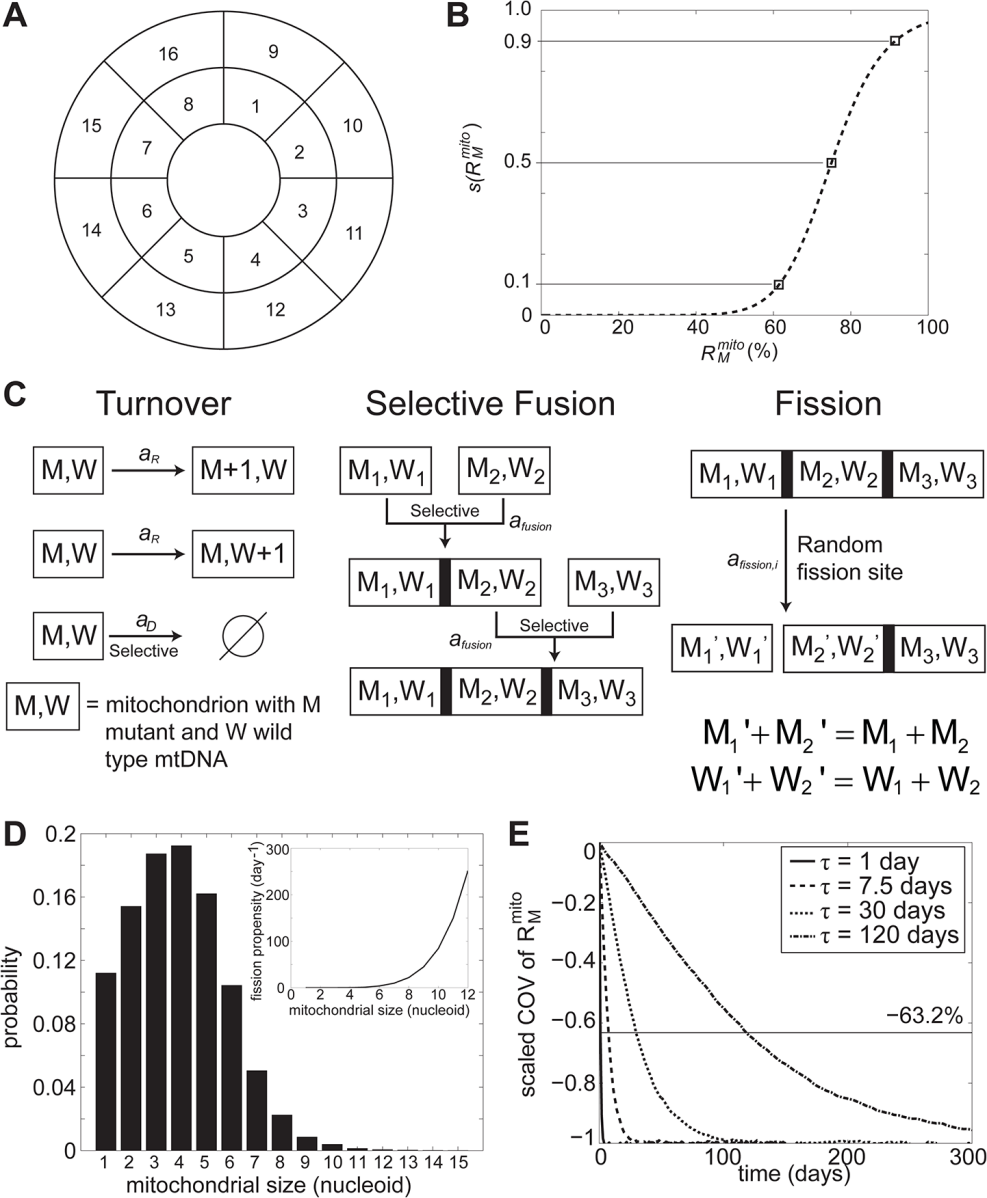
Detailed model implementation. (A) Partitioning of the 2D circular cell in the model. (B) OXPHOS defect function $s\left(R_{M}^{mito}\right)$. (C) Schematic diagram of model implementation of mitochondrial turnover and fusion-fission (see text for detailed explanation). (D) Steady state distribution of mitochondrial nucleoid contents. The inset shows the fission propensity as a function of mitochondrial nucleoid content. (E) Nucleoids mixing time. Mitochondrial heterogeneity in each cell is represented by the mean coefficient of variation (COV) of *R*_*M*_^*mito*^. The mean COV of R_M_^mito^ is scaled such that the steady state value is −100%. The results come from simulating only mitochondrial fusion-fission (without mitochondrial turnover) for 10,000 cells.

Fig 1 provides a summary of the mitochondrial QC model. In general, the model describes mitochondrial turnover and fusion-fission processes. Extending our previous model [42], we accounted for the selectivity of mitochondrial fusion and mitophagy (see box in Fig 1), where mitochondria with OXPHOS defects (resulting in lowered membrane potential) are prevented from fusing with other mitochondria and are preferentially degraded. Briefly, we tracked the numbers of WT and mutant mtDNA nucleoids in individual mitochondria organelles of a cell. Here, we assumed a circular cell, partitioned into multiple subcellular compartments (see Fig 2*A*). However, the choice of the cellular shape and size was not critical to the outcome of the model simulations (see S1 Fig). We modeled mitochondria organelles as separate entities, each defined by its nucleoid content. Because of mitochondrial fusion, these entities could possess subcompartments, reflecting the nucleoid contents of the parental fusing mitochondria (see Fig 2*C*). In the model simulations, the number of mitochondria in a cell and the nucleoid content of each mitochondrion vary with time due to two key processes: mitochondrial turnover (mitophagy and mitogenesis) and the fusion-fission process.

We simulated the replication of mtDNA nucleoids with retrograde signaling, in which the propensity (rate) of replication increased with the average fraction of mutant mtDNA in the mitochondrial population. We adopted the “faithful nucleoid” model [43], where nucleoids were replicated to produce identical daughters and there was no exchange of mtDNA molecules among nucleoids. As mentioned above, we implemented mitophagy and fusion to be selective. To do so, we explicitly described the effect of mtDNA mutations on OXPHOS capacity at the level of single mitochondrion (see Fig 2*B*). Meanwhile, we simulated mitochondrial fusion events by combining the subcompartments of the parental mitochondria entities. Conversely, in fission events involving a mitochondrion with subcompartments, we formed two daughter mitochondria by splitting the parent mitochondrion at one of the partition sites (called fission sites), and exchanged mtDNA nucleoids randomly between the previously adjoining subcompartments (see Fig 2C). For a mitochondrion without any subcompartment, we created a new mitochondrion during a fission event, and transferred a random number of nucleoids from the first mitochondrion.

We also tracked the spatial distribution of mitochondria across the subcellular compartments. We assumed that the movements of mitochondria across compartments occur only as a consequence of mitochondrial fusion-fission process, i.e. mitochondrial motility that is independent of fusion-fission is inconsequential in the context of mitochondrial QC. This assumption is based on the observation that mitochondrial motility is abolished when mitochondrial fusion is inhibited [28], suggesting that mitochondrial fusion and motility are closely coupled. More details regarding the model are provided in Methods.

We formulated the model using the framework of chemical master equation (CME) [44]. This approach was chosen in order to describe the stochasticity in the mitochondrial turnover and fusion-fission process involving a (small) population of mitochondria and of mtDNA nucleoids in a cell. Evidence suggests that there indeed exists a significant stochastic factor influencing the clonal expansion of mtDNA mutations [45]. The propensity functions used for mitochondrial turnover and fusion-fission are detailed in Mitochondrial Fusion-Fission in Methods, and the nominal model parameters are given in Table 1. For the purpose of the study, we simulated the clearance or accumulation of mutant mtDNA nucleoids from a single (*de novo*) mutant molecule in a cell. As a measure of clonal expansion, we calculated the average fraction of mutant mtDNA per cell, denoted by ${\bar{R}}_{M}^{cell}$, over a population of 10,000 simulated cells. Unless stated otherwise, the model simulations started with a premixing step in which we implemented only the mitochondrial fusion-fission process (without the mitochondrial turnover) until the nucleoid distribution among mitochondria and the spatial distribution of mitochondria in the cell reached steady-state (see Premixing Simulations in Methods). The purpose of the premixing step was to remove any dependence of the model prediction on the (unknown) initial distribution of mitochondria and mtDNA. We carried out the model simulations using the stochastic simulation algorithm [46] for a duration of 300 days, within which a single mutant nucleoid was cleared in simulations using the nominal model parameters in Table 1.

**Table 1:** Nominal model parameter values.

Parameter	Value	Remarks
**OXPHOS Defect Function**		
***K***	75%	Based on the experimentally observed range of mtDNA mutation burden associated with loss of cellular respiratory capacity; between 60% to 90% [10, 22, 60].
***m***	11.0	Manually adjusted such that *s*(60%) = 0.1 and *s*(90%) = 0.9 [10, 22, 60]. See also Fig 2*B*.
**Mitophagy**		
***k***_***D***_	0.023 day^-1^	There exist discrepancies in the reported half-lives of mitochondria, ranging from 2 days to 2 years [76–80]. We have previously suggested that a turnover rate in the order of months is consistent with published mtDNA mutation levels [81]. Here, we used a half-life of 30 days for *k*_*D*_.
***r***_***D*,*max***_	5	Based on an experimental observation where a depolarized mitochondrion was removed by mitophagy in 1–8 hours, but mitochondrion with a normal membrane potential was only removed in 5–24 hours [15].
**mtDNA Replication**		
***a***_***R*,*0***_	7.4 day^-1^	Calculated by multiplying *k*_*D*_ with the steady state number of nucleoids of 320. Here, the replication of WT nucleoid balanced their removal by mitophagy. The steady state nucleoid number was determined by assuming an average number of 80 mitochondria per cell [74] and 4 nucleoids per mitochondrion [72].
***r***_***R*,*max***_	9	Based on the observed increase in mtDNA copy number, between 2 to 20 fold, accompanying clonal expansion of mutant mtDNA [60, 82–84]. We chose an intermediate value of 10 fold.
**Mitochondrial fusion (τ = 7.5 days)**		
***a***_***fusion*,*0***_	0.123 mitochondrion^−1^ day^−1^	Manually adjusted to give a nucleoid mixing time of *τ* = 7.5 days (see Fusion-Fission Parameters in Methods)
***r***_***fusion*,*max***_	80%	Based on an experimental observation showing that depolarized mitochondria were 6 times less likely to undergo fusion than mitochondria with normal membrane potential [15].
**Mitochondrial fission (τ = 7.5 day)**		
***V***_***F*,*max***_	8.6 × 10^4^ day^−1^	Manually adjusted based on nucleoid distribution among mitochondria
***K***_***F***_	30	
***n***	6.0	

### Global Sensitivity Analysis

Parametric sensitivity analysis (PSA) is a systems analysis commonly used for mapping the dependence of biological system behavior on its model parameters [47]. In this analysis, we evaluate sensitivity coefficients, quantifying how much a perturbation in the model parameter affects the behavior of the model. We use the sensitivity coefficients as a relative measure of the importance of parameters, and to rank parameters and the associated biological processes based on their relative impact on system behavior. Parameters with high sensitivity magnitudes correspond to processes that are most critical for the model output. Two types of sensitivity analyses exist, local and global, depending on the nature of the parameter perturbations [47]. A local PSA uses infinitesimal parameter perturbations around well-known parameter value and thus the analysis is typically applied when the uncertainties in the model parameter values are small. Meanwhile, a global sensitivity analysis (GSA) involves finite (large) parameter perturbations within a range of values, and thus, such analysis is appropriate when the parameter values have relatively large uncertainties.

We performed the GSA to elucidate how much ${\bar{R}}_{M}^{Cell}$ depend on the parameters of mitochondrial QC process. More specifically, we calculated the first and second order sensitivity coefficients with respect to each model parameter and every pairwise parameter combination using variance-based global sensitivity coefficients (see Global Parametric Sensitivity Analysis in Methods). The ranges of parameter values are provided in Table 2. The first order sensitivities *S*_*i*_ (*t*) reflect the main effect of the parameter *p*_*i*_ on ${\bar{R}}_{M}^{Cell}\left(t\right).$ The second order sensitivities *S*_*ij*_ (*t*) represent the joint effect of the parameters *p*_*i*_ and *p*_*j*_ on ${\bar{R}}_{M}^{Cell}\left(t\right)$, excluding the main effect of each individual parameter. The second order sensitivity coefficients point to important interactions between two parameters or factors [47]. Here, we used the sensitivity coefficients to rank the parameters according to their effects on ${\bar{R}}_{M}^{Cell}$, in order to understand the relative impacts of different mitochondrial QC processes on clonal expansion.

**Table 2:** Range of parameter values for global sensitivity analysis.

Parameter	Value range	Remarks
**Replicative advantage (*k***_***R***_ **)**	1–2	The replication propensity of mutant mtDNA is multiplied by this factor to simulate replicative advantage.
**Fusion-fission rate (*τ*)**	7.5–30 days	Different mixing time constants are achieved by simultaneously scaling the parameters *a*_*fusion*_ and *V*_*F*,*max*_ with equal ratios. The range is consistent with a four-fold decline in the fusion-fission rate in aged human endothelial cells [52].
**Mitophagy rate (*k***_***D***_ **)**	0.0069–0.023 day^-1^	The range corresponds to mitochondrial half-life (*t*_*1/2*_) of 30–100 days.
**Mitophagy selectivity strength (*r***_***D*,*max***_ **)**	1–5	
**Fusion selectivity (*r***_***fusion*,*max***_ **)**	0–80%	
**Retrograde signaling (*r***_***R*,*max***_ **)**	5–10	
**OXPHOS defect threshold (*K***_***D***_ **, *K***_***fusion***_ **, *K***_***R***_ **)**	60–90%	

### Sensitivity Analysis of Mitochondrial Quality Control

We calculated the first and second order global sensitivity coefficients of the model as described above, and ranked the parameters and pairs of parameters based on the magnitude of the sensitivities (the first rank corresponding to the largest magnitude). Table 3 provides a list of the important parameter ranking including the average rank over different time points (for the complete list, see S1 Table). The average ranking suggested that the clonal expansion of mtDNA mutations is most highly sensitive to the following parameters (in decreasing rank): replicative advantage (*k*_*R*_), mitophagy selectivity strength (*r*_*D*,*max*_), fusion selectivity strength (*r*_*fusion*,*max*_), mitophagy selectivity threshold (*K*_*D*_), and fusion-fission frequency. The existence of replicative advantage (RA) of mutant mtDNA, where mutant molecules have higher propensity to replicate than WT (see Mitochondrial DNA Replication in Methods), was the most important determining factor of whether or not a mutant mtDNA molecule would clonally expand. This finding is in agreement with intuition, but the degree of sensitivity is nonetheless insightful. Interestingly, mitochondrial half-life (related to the parameter *k*_*D*_) was ranked lower than the selectivity of mitophagy (see S1 Table). Similarly, parameters associated with the selectivity of fusion were ranked higher than the rate of fusion-fission.

**Table 3:** Ranking of mitochondrial QC processes by global sensitivity analysis.

Parameters	Day 50	Day 100	Day 150	Day 200	Day 250	Average Rank
**Replicative advantage (*k***_***R***_ **)**	1	1	1	1	1	1
**Mitophagy selectivity strength (*r***_***D*,*max***_ **)**	2	2	2	2	2	2
**Fusion selectivity strength (*r***_***fusion*,*max***_ **)**	3	3	3	3	3	3
**Mitophagy selectivity threshold (*K***_***D***_ **)**	4	4	4	4	4	4
**Fusion-fission rate (*τ*)**	7	5	5	5	6	5.6
**Replicative advantage (*k***_***R***_ **) + Mitophagy rate (*k***_***D***_ **)**	5	6	6	7	10	6.8
**Mitophagy rate constant (*k***_***D***_ **) + Mitophagy selectivity threshold (*K***_***D***_ **)**	6	7	9	10	12	8.8
**Replicative advantage (*k***_***R***_ **) + Mitophagy selectivity strength (*r***_***D*,*max***_ **)**	21	9	8	6	5	9.8
**Fusion selectivity threshold (*K***_***F***_ **)**	8	10	12	11	13	10.8
**Replicative advantage (*k***_***R***_ **) + Fusion-fission rate**	19	11	10	9	8	11.4

On the other hand, nuclear retrograde signaling was consistently ranked low, indicating a process of low importance. The low sensitivity of mtDNA mutant expansion with respect to retrograde signaling is perhaps expected as the retrograde response is activated only when mutant mtDNA molecules have already accumulated to a high enough level and thus have little effect on the clearance of new mtDNA mutations. Nevertheless, retrograde signaling could retard the clonal expansion of mtDNA by indirectly reducing stochasticity [38], an effect that depends in non-trivial ways on fusion-fission rate [42]. Thus, the GSA of our model suggested that among mitochondrial QC processes, mitochondrial fusion-fission, selective mitophagy, and selective fusion are the most relevant targets for preventing the clonal expansion of mtDNA mutations.

### Mitochondrial Fusion-Fission Trade Off

To gain a deeper understanding of the role of individual mitochondrial QC processes in clearing *de novo* mutant mtDNA, we simulated the model under different perturbations of model parameters. When investigating the effects of varying fusion-fission frequency, we multiplied the propensities of fusion and fission by the same factor (>1 to increase or <1 to lower the fusion-fission frequency). By doing so, the ratio between the number of fusion and fission events per unit time will stay the same. In the following, we labeled the simulations from different fusion-fission frequencies using the corresponding mixing time *τ* (see Fig 2*E* and Fusion-Fission Parameters in Methods). Model simulations using the nominal parameter values in Table 1 correspond to a mixing time of *τ* = 7.5 days. A lower *τ* indicates more frequent fusion-fission, and vice versa, a higher *τ* means less frequent fusion-fission.

**Fig 3:**
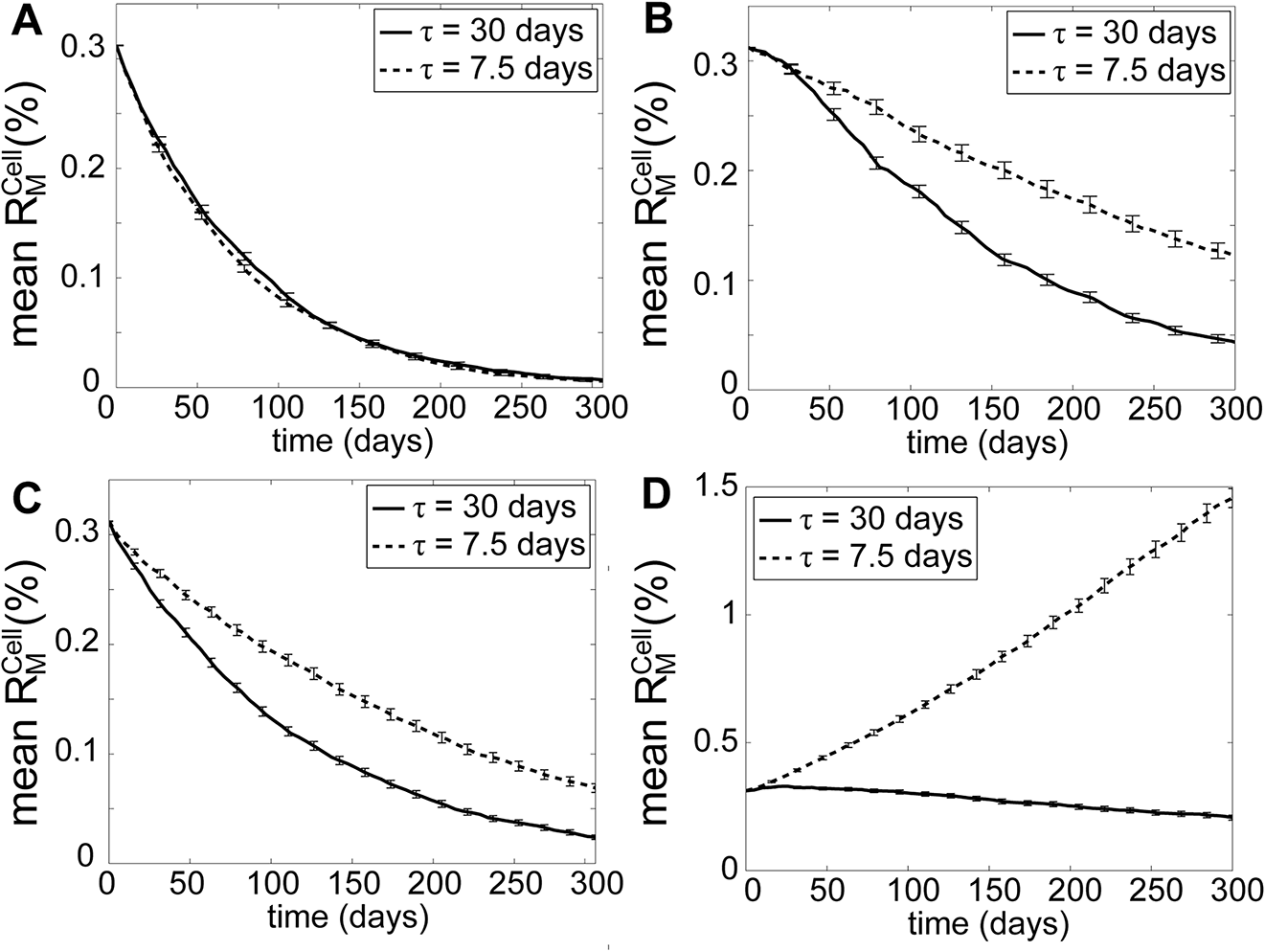
Model simulations with and without selectivity in mitochondrial fusion. (A & B) Model simulations under two different mixing time constants (*τ* = 7.5 and 30 days) for (A) mutations without RA and (B) mutations with RA (*k*_*R*_ = 2). (C & D) Model simulations of non-selective fusion for (C) mutations without RA and (D) with RA. The error bars represent the standard deviation of ${\bar{R}}_{M}^{Cell}\left(t\right)$ among 10,000 cells.

Model simulations as shown in Fig 3*A* and 3*B* suggested that the mitochondrial QC can effectively remove a single *de novo* mtDNA mutation, as indicated by the steady decrease in ${\bar{R}}_{M}^{Cell}$ for *τ* = 7.5 days, even when mutant mtDNA molecules possessed a RA. Here, a zero ${\bar{R}}_{M}^{Cell}$ means that every cell in the population harbors homoplasmic WT mtDNA. In *C*. *elegans*, the clearance of UVC-induced mtDNA lesions has been shown to decay following a similar time profile [29]. In the absence of selectivity of mitochondrial fusion, mitochondrial QC could still remove *de novo* mutant mtDNA without RA (see Fig 3*C*). Meanwhile, mutant mtDNA with RA was cleared in simulations with *τ* = 30 days, but not in simulations with *τ* = 7.5 days (see Fig 3*D*). In general according to the simulations in Fig 3 less frequent fusion-fission has a beneficial effect on the clearance of *de novo* mtDNA mutations.

**Fig 4:**
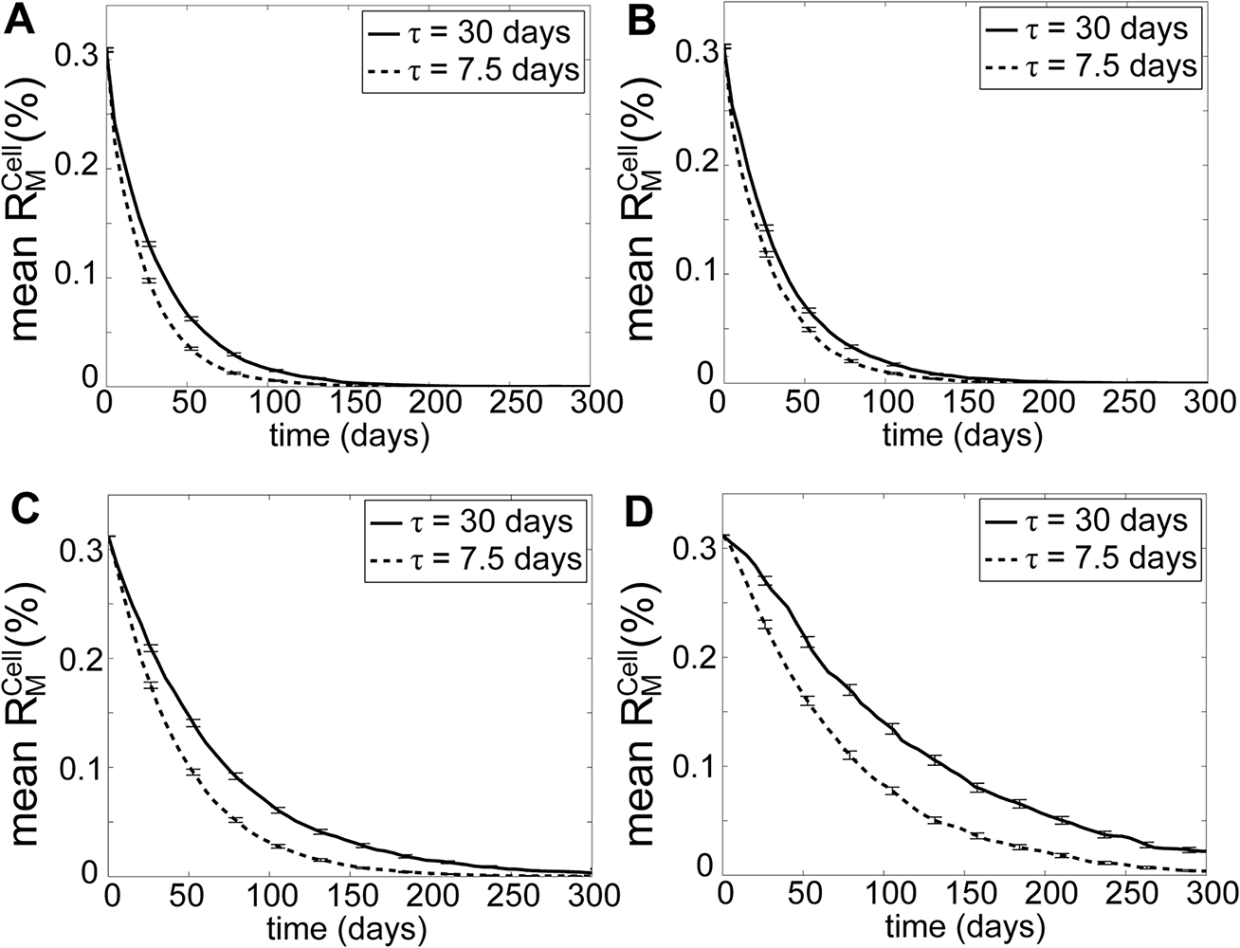
High selectivity in mitochondrial fusion and mitophagy providing efficient removal of mutant mDNA. (A & B) Model simulations with high mitophagy selectivity (*r*_*D*,*max*_ = 199) and non-selective fusion for (A) mutations without RA and (B) with RA (*k*_*R*_ = 2). (C & D) Model simulations with high fusion selectivity (*r*_*fusi*,*max*_ = 100%, *r*_*D*,*max*_ = 5) for (C) mutations without RA and (D) with RA. The error bars represent the standard deviation of ${\bar{R}}_{M}^{Cell}\left(t\right)$ among 10,000 cells.

**Fig 5:**
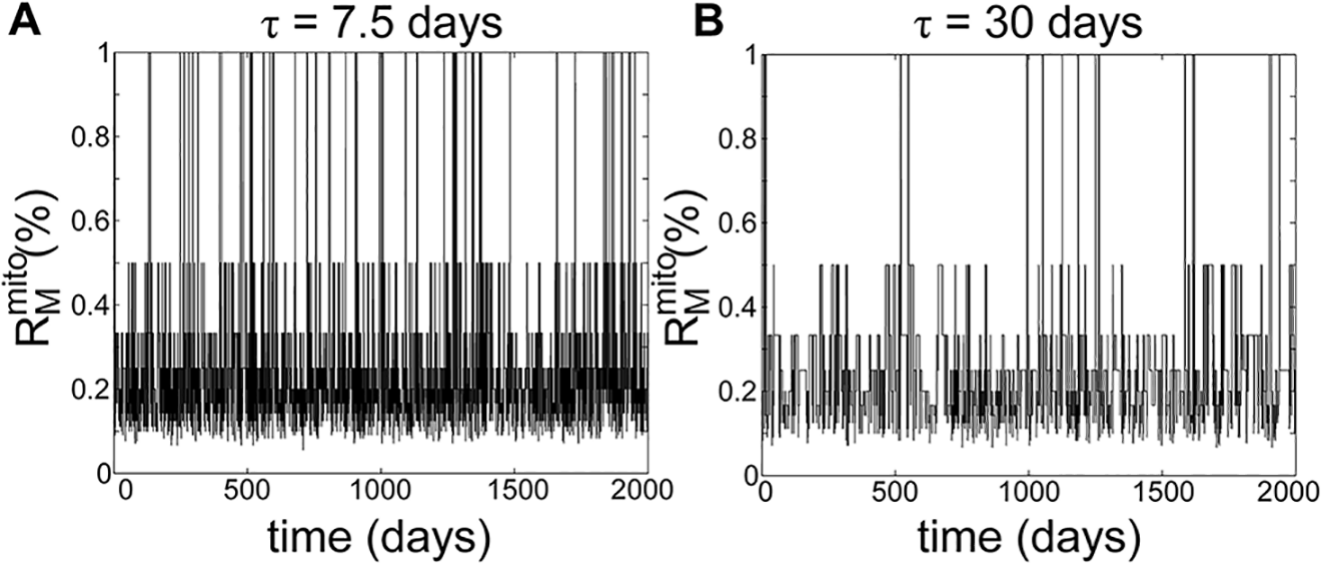
Appearance and disappearance of mutant-rich mitochondria. The simulations were performed with only mitochondrial fusion-fission process and with non-selective fusion, for (A) *τ* = 7.5 days and (B) *τ* = 30 days.

We observed the opposite effect of fusion-fission frequency on mutant mtDNA clearance when mitophagy selectivity was significantly increased in the absence of fusion selectivity (see Fig 4*A* and 4*B*). In this case, more frequent fusion-fission quickened the removal of mutant mtDNA regardless of RA. The reversal of trend can be explained by considering the time scales of mitochondria harboring a high fraction of mutant mtDNA, which we refer to as *mutant-rich mitochondria*. Mutant-rich mitochondria can arise randomly during fissions, and disappear by fusing with other mitochondria. To illustrate this phenomenon, we simulated the model with only the fusion-fission process in a cell containing a single mutant mtDNA nucleoid (in the absence of mitochondrial turnover and selectivity of fusion). Fig 5 shows the fractional mutation burden *R*_*M*_^*mito*^ of the mitochondrion harboring the mutant nucleoid, illustrating the appearance and disappearance of mutant-rich mitochondria. When fusion-fission became more frequent (i.e. for lower *τ*), mutant-rich mitochondria occurred more frequently (4.1×10^–4^ day^-1^mitochondrion^-1^ for *τ* = 7.5 days vs. 9.5x10^-5^ day^-1^mitochondrion^-1^ for *τ* = 30 days). However, with more frequent fusion-fission, these mutant-rich mitochondria had a shorter average lifetime (0.9 days for *τ* = 7.5 days vs. 3.7 days for *τ* = 30 days). Nevertheless, the fraction of time that the mtDNA mutant molecule existed in a mutant-rich mitochondrion did not change, since the numbers of fusion and fission events remained at the same ratio. Therefore, while a quicker fusion-fission process could produce more mutant-rich mitochondria, the shorter existence of these mitochondria meant that there was less time for selective mitophagy to remove them. If the time scale of mitophagy was much longer than the duration of these mutant-rich mitochondria, then mutant mtDNA molecules would get diluted among the population of mitochondria, preventing the clearance of such molecules.

In corollary, prolonging the presentation of mutant-rich mitochondria, for example by increasing the selectivity of fusion, should improve the removal of such mitochondria by mitophagy. Simulation results clearly showed that when mutant-rich mitochondria were strongly prohibited from undergoing mitochondrial fusion (by setting *r*_*fusion*,*max*_ = 100%), increasing fusion-fission rate again enhanced the clearance of mtDNA mutations (see Fig 4*C* and 4*D*). The faster removal of mutant nucleoids here was a consequence of more frequent generation of mutant-rich mitochondria, their subsequent isolation by selective fusion and removal by selective mitophagy. However, under a milder selectivity of fusion (*r*_*fusion*,*max*_ = 80%), more frequent fusion-fission did not have a significant impact on the removal rate of mutant mtDNA, and even caused a slower clearance of mutant mtDNA when RA was considered as mentioned earlier (see Fig 3*B* and 3*D*). This result again demonstrated the importance of prolonging the duration of mutant-rich mitochondria for an effective removal of mutant mtDNA molecules.

Taken together, our model simulations illustrate the trade-off in the actions of mitochondrial fusion-fission process in the context of *de novo* mtDNA mutation removal. On the one hand, the fission process is advantageous in generating mutant-rich mitochondria, which allows phenotypic expression of mtDNA mutations in the corresponding mitochondrion and its preferential removal by mitophagy. On the other hand, the fusion process dilutes mutant mtDNA among the WT population in the cell. While more frequent fusion-fission events increase the occurrence of mutant-rich mitochondria, these mitochondria exist for a shorter period of time. The benefit of faster fusion-fission in removing mutant mtDNA molecules therefore depends on the efficiency by which mitophagy can detect and remove the aforementioned mutant-rich mitochondria.

### Impacts of a Decline in Mitochondrial Quality Control

Our model simulations as discussed above showed that the mitochondrial QC can effectively clear mutant mtDNA from the population. However, the fact that mutant mtDNA do clonally expand with ageing suggests the existence of a failure or deterioration in one or more QC mechanisms. Drift in the gene expression level has been observed during ageing [48]. The expression of mitochondrial genes and genes associated with mitochondrial energy production has been observed to drop with age in mice, rats and humans [48]. Moreover, the abundance of mitochondrial proteins can decline by as much as 50% in older individuals [49]. At the same time, genes related to mitochondrial QC, for example PINK1 (involved in the targeting of depolarized mitochondria for mitophagy), are also downregulated in age-related diseases, such as in neurons of Parkinson’s disease patients [50]. A recent mathematical modeling cum experimental study showed that mitochondrial fusion-fission occur less frequently in senescent cells despite of being still tightly coupled [51]. While it is not known if the age-related decline in the associated proteins is the cause of mitochondrial QC deterioration, it is still instructive to understand the implications of a general decline in mitochondrial QC with age.

**Fig 6:**
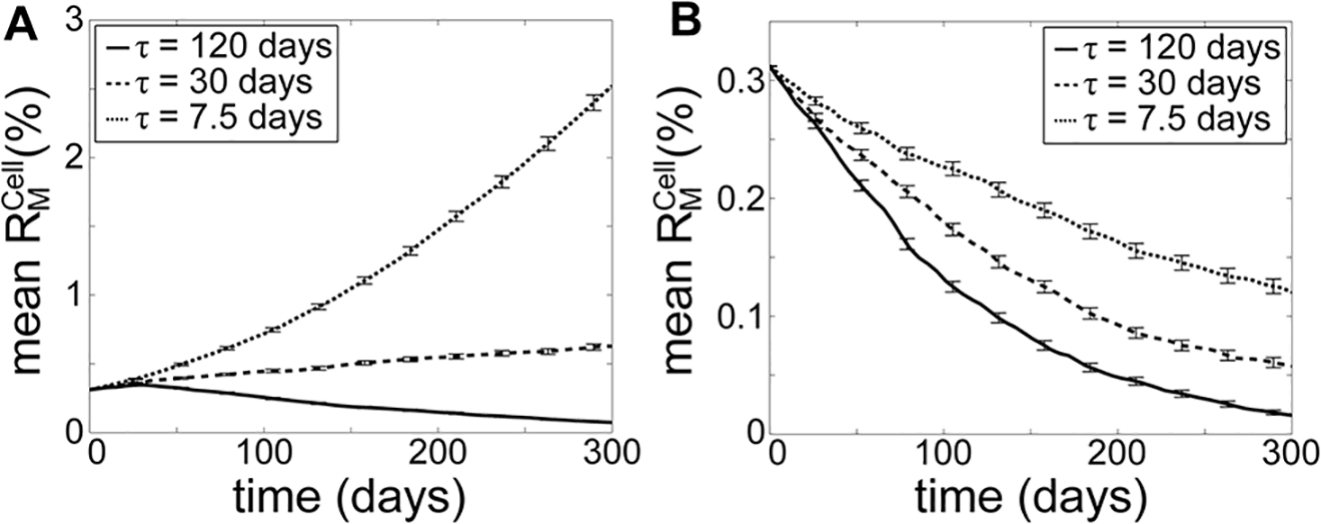
Effects of lower selectivity of mitochondrial fusion and mitophagy. Model simulations were performed by lowering the mitophagy selectivity (*r*_*D*,*max*_) and fusion selecitivity (*r*_*fusi*,*max*_) to half (50%) of the nominal values, for mutant mtDNA molecules (A) with RA (*k*_*R*_ = 2) and (B) without RA. The error bars show the standard deviation of ${\bar{R}}_{M}^{Cell}\left(t\right)$ among 10,000 cells.

To this end, we performed model simulations where the values of mitophagy selective strength *r*_*D*,*max*_ and fusion selective strength *r*_*fusion*,*max*_ were each lowered by 50%. Under such changes, mitochondrial QC could no longer remove mutant mtDNA molecule with RA (by comparing Fig 3*B* with Fig 6*A* for *τ* = 7.5 and 30 days). When the fusion-fission rate remained at the nominal value (*τ* = 7.5 days), mutant mtDNA with RA quickly expanded, as selective mitophagy could not remove mutant-rich mitochondria quickly enough before they disappeared. Interestingly, when the decline in the selectivity was accompanied by a significant drop in the fusion-fission frequency, the mutant molecules could still be cleared, albeit slowly (see Fig 6*A*, *τ* = 120 days). Meanwhile, the removal rate of mutant mtDNA without a RA slowed down with lower selectivity of fusion and mitophagy (compare Fig 3*A* with Fig 6*B*). Therefore, less frequent fusion-fission could provide a protective effect on mtDNA integrity against declining selectivity of mitochondrial fusion and mitophagy.

## Discussion

Based on the model simulations above and the results of GSA, age-related (clonal) expansion of mtDNA mutations likely involves deterioration in either the selectivity of mitophagy or that of mitochondrial fusion or both, i.e. the mechanisms that are responsible in the isolation and targeted removal of mutant mtDNA molecules. Under this condition, slower mitochondrial fusion-fission, which is also expected to happen with age [51, 52], may actually retard the accumulation of mutant mtDNA. Our model simulations offered an alternative explanation for the benefit of decelerating fusion-fission with age, without assuming the spread of infectious damage among mitochondria due to fusion-fission [40].

Because of the role of mitochondrial fusion-fission in maintaining normal mitochondrial function, as well as its relevance in mitochondrial diseases, this process has been suggested as a promising target for therapeutic treatment in the context of age-related diseases [53, 54]. In particular, senescent cells have been observed to suffer from low activity of mitochondrial fusion-fission, which has been suggested as the cause for the accumulation of dysfunctional mitochondria in these cells [52]. The decline in mitochondrial fusion-fission has also been associated with sarcopenia, neurodegenerative disease and the ageing process itself [27, 55]. Naturally, reversing the age-related change in mitochondrial fusion-fission rate may at first seem reasonable. However, according to the model simulations above, the success of this intervention will sensitively depend on the efficiency of selective mitophagy. An upregulation of mitochondrial fusion-fission in aged cells without a concurrent improvement in the selective mitophagy may aggravate the accumulation of mutant mtDNA molecules and worsen mitochondrial functionality. Any intervention aimed at improving or boosting mitochondrial QC mechanisms will therefore need to consider the balance and interactions among the processes involved, most importantly the selective mitophagy and mitochondrial fusion-fission. Experimental evidence from our laboratory [56] and others [57] has shown modest improvement in mitochondrial function when mitochondrial turnover is increased. However, model simulations and global sensitivity analysis in this study suggest that the selectivity of mitophagy and fusion, not their rates, would be the more interesting targets of slowing down clonal expansion of mtDNA mutations and the corresponding decline of mitochondrial function with age.

In summary, simulations of the mathematical model presented in this study illustrated the intricate interplay among the processes involved in the mitochondrial quality control and its implications on the clearance of mutant mtDNA molecules. Our model also produced an interesting insight regarding the dependence of mitochondrial QC on the lifetime of mutant-rich mitochondria that are transiently created by stochastic mixing of mtDNA molecules through mitochondrial fusion-fission. While faster mitochondrial fusion-fission creates more mutant-rich mitochondria, these mitochondria however have shorter lifetimes. Therefore, upregulating mitochondrial fusion-fission is beneficial in removing mutant mtDNA only when mutant-rich mitochondria could be efficiently isolated and removed by the coupled processes of selective mitochondrial fusion and mitophagy. Global sensitivity analysis of the model further confirmed the selectivity of fusion and mitophagy as the most important parameters for clearing mutant mtDNA. Our results thus suggested that the decline of these selective processes is a possible cause of expansion of mutant mtDNA molecules. In this regard, slower fusion-fission could actually lessen the negative effect of such decline on mtDNA integrity.

## Methods

The mitochondrial QC model describes the time evolution of mitochondria organelles and mtDNA nucleoid distribution in individual mitochondria. In the following, we denote the number of WT nucleoids in the *i*-th mitochondrion as *W*_*i*_ and the number of mutant nucleoids as *M*_*i*_. The fraction of mutant nucleoids in the *i-*th mitochondrion is defined as *R*_*M*,*i*_^*mito*^ = *M*_*i*_ / (*W*_*i*_ + *M*_*i*_), while the fraction of mutant nucleoids in a cell is defined as $R_{M}{}^{cell}=\sum_{i}M_{i}/\sum_{i}\left(W_{i}+M_{i}\right)$. An *R*_*M*_^*cell*^ value of 0 or 1 thus indicates a cell harboring homoplasmic WT or mutant mtDNA, respectively. To account for the dynamic spatial distribution of mitochondria in the cell, we divide each cell into multiple compartments, as shown in Fig 2*A*. Following our previous study [42], we use a circular cell with a diameter of 20 μm [58] and with subcellular compartments of approximately 3 μm wide. The size of the cellular compartments reflects the maximum length of directed movements of mitochondria [59].

In the model, we consider the following processes: mtDNA replication, mitophagy, mitochondrial fusion and mitochondrial fission. We formulate the model using the well-established CME framework. More specifically, for each of the above processes, we define a propensity function *a*_*j*_ such that the probability of the *j-*th process to take place in a time window *dt* is given by *a*_*j*_ *dt*. Below, we provide the propensity functions and the rationale and assumptions that we have taken to derive them. We also describe the implementation of the global sensitivity analysis. Finally, the model parameter values and their justifications are given in Table 1.

### OXPHOS Defect due to Mitochondrial DNA Mutations

Here, we consider deleterious mtDNA mutations that cause functional defects in OXPHOS. However, because of mitochondrial heteroplasmy and complementation by WT mtDNA, the energetic impact of the mtDNA mutations will only become apparent when the fraction of mutant mtDNA population exceeds a critical threshold [10]. We choose a sigmoidal function to describe the threshold effect of mtDNA mutations on OXPHOS capacity of a mitochondrion. This choice is supported by an experimental observation showing a sigmoidal relationship between mitochondrial function and mutation load [60]. We formulate the OXPHOS defect function using a standard sigmoidal family of function:

$$s\left(R_{M}^{mito}\right)=\frac{{\left(R_{M}^{mito}\right)}^{m}}{K^{m}+{\left(R_{M}^{mito}\right)}^{m}}.$$

The value $s\left(R_{M}^{mito}\right)\in \left[0,1\right]$ reflects the degree of OXPHOS defects, where a value of 0 corresponds to no OXPHOS defect (at $R_{M}^{mito}=0$) and a value of 1 corresponds to completely dysfunctional OXPHOS. Here, the parameter *K* represents the midpoint of the threshold effect (i.e. *s* (*K*) = 0.5), while the parameter *m* changes the width (sharpness) of the threshold region (see Fig 2*B*). The values of *K* and *m* used in this study are provided in Table 1. Fig 2*B* shows $s\left(R_{M}^{mito}\right)$ with parameter *K* of 75%. The width of $s\left(R_{M}^{mito}\right)$ is here defined as the range of *R*_*M*_^*mito*^ corresponding to *s =* 0.1 and *s* = 0.9.

### Mitophagy

We formulate the propensity of mitophagy of the *i-*th mitochondrion as the product of the basal mitophagy rate *k*_*D*_ and the selectivity function of mitophagy, according to:

$$a_{D,i}\left(R_{M,i}^{mito}\right)=k_{D}\left(r_{D,max}s\left(R_{M,i}^{mito}\right)+1\right)$$

In the model, mitophagy involves removing a single mitochondrion from the cell (see Fig 2*C*). The parameter *k*_*D*_ is related to the half-life of mitochondrial DNA *t*_1/2_, specifically *k*_*D*_ = ln(2) / *t*_1/2_. The selectivity function $\left(r_{D,max}s\left(R_{M,i}^{mito}\right)+1\right)$ becomes larger than 1 for mitochondria with OXPHOS defects, i.e. mitochondria with $s\left(R_{M,i}^{mito}\right)$ > 0. Thus, the likelihood of dysfunctional mitochondria to be removed by mitophagy will be higher than that of functional mitochondria. The parameter *r*_*D*,*max*_ is related to the degree of mitophagy selectivity, where *r*_*D*,*max*_ + 1 gives the maximum amplification of mitophagy propensity. For example, mitochondria with fully dysfunctional OXPHOS (with $s\left(R_{M}^{mito}\right)=1$) are autophagosized at a propensity of (*r*_*D*,*max*_ + 1) times higher than functional mitochondria (with $s\left(R_{M}^{mito}\right)=0$). Note that for mitochondria with $s\left(R_{M}^{mito}\right)=0$, the mitophagy propensity assumes the baseline value *a*_*D*,*i*_ = *k*_*D*_.

### Mitochondrial DNA Replication

Under normal conditions, mitochondrial DNA content in a cell is tightly regulated [61]. Defects in OXPHOS due to mtDNA mutations can trigger mitochondrial retrograde signaling that in turn upregulates mitogenesis and mtDNA replication [1]. In mitochondrial myopathies for example, a significant percentage of mtDNA are deleterious and mitochondrial mass and mtDNA copy number have both been shown to increase [62–64]. To reflect this relationship in our model, we calculate the mtDNA nucleoid replication propensity by multiplying the basal propensity of replication *a*_*R*,0_ with a retrograde response function, such that an increase in the fraction of dysfunctional mitochondria will lead to increased mitochondrial biogenesis. This implementation results in behavior that is in accordance with experimental observation [60]. Overall, we formulate the replication propensity as follows:

$$a_{R}\left({\bar{R}}_{M}^{mito}\right)=k_{R}a_{R,0}\left(r_{R,max}s\left({\bar{R}}_{M}^{mito}\right)+1\right).$$

The number of WT or mutant mtDNA (*W* or *M*, respectively) is increased by 1 in a nucleoid replication event (see Fig 2*C*). In the absence of any mutant mtDNA, the nucleation propensity reduces to the basal replication rate *a*_*R*,0_. However the presence of mutant mtDNA will trigger the retrograde response, at a degree that varies with the average fraction of mutant mtDNA among mitochondria in the cell. This formulation is motivated by studies of Sarcopenia, where hyperproliferation of mtDNA mutant molecules also leads to OXPHOS dysfunction in muscle fibres despite having a relatively stable WT mtDNA population [62]. Finally, the maximum amplification of nucleoid replication by the retrograde response occurs when $s\left({\bar{R}}_{M}^{mito}\right)=1,$ corresponding to a propensity of replication $a_{R}\left({\bar{R}}_{M}^{mito}\right)=k_{R}a_{R,0}\left(r_{R,max}+1\right)$.

The parameter *k*_*R*_ is used to implement replicative advantage of mutant mtDNA. Many experimental observations reported in the literature suggest the presence of RA of mutant mtDNA, especially for mtDNA with large deletion mutations. For example, in experiments, mtDNA molecules with larger deletions have been shown to be able to re-populate a cell much faster than WT and mutant mtDNA with shorter deletions [65–67]. These observations have subsequently been used to support the hypothesis that mutant mtDNA with large deletions are replicated preferentially or faster than WT mtDNA. While the molecular mechanism of such RA is not precisely known, a recent study has attributed this advantage to a local feedback between the transcription and replication of mtDNA with a deletion mutation [68]. In the model simulations with RA, the parameter *k*_*R*_ was set to a value larger than 1 for mutant mtDNA, while *k*_*R*_ was set to 1 for WT mtDNA. In simulations without replicative advantage, *k*_*R*_ = 1 was used for all mtDNA.

### Mitochondrial Fusion-Fission

#### Fusion

As mentioned earlier, mitochondrial motility is coupled with mitochondrial fusion. Since the size of cellular compartments reflected the maximum distance of directed movements of a single mitochondrion, mitochondrial fusion in our model can only occur between two mitochondria that reside in the same or adjacent cell compartments. As an extension of our previous model [42], mitochondrial fusion is simulated as a selective process, where mitochondria with OXPHOS defects have a lower propensity to undergo fusion. We calculate the propensity of fusion between any feasible pairs of mitochondria (e.g. the *i-*th and *j*-th mitochondria) according to:

$$a_{fusion,\left(i,j\right)}(R_{M,i}^{mito},R_{M,j}^{mito})=a_{fusion,0}r(R_{M,i}^{mito})r(R_{M,j}^{mito})$$

where the function $r(R_{M}^{mito})$ describes the selectivity of mitochondrial fusion as follows:

$$r(R_{M}^{mito})=1-r_{fusion,max}s\left(R_{M}^{mito}\right)$$

In this case, mitochondria with OXPHOS defects (i.e. $s\left(R_{M}^{mito}\right)>0$) will have $r(R_{M}^{mito})<1$, and are therefore less likely to participate in a fusion process. The parameter *r*_*fusion*,*max*_ gives the maximum relative decrease of fusion propensity, and therefore describes the degree of selective fusion. For example, by setting *r*_*fusion*,*max*_ = 1, a mitochondrion with fully dysfunctional OXPHOS (i.e. $s\left(R_{M}^{mito}\right)=1$) will be completely prevented from fusion since the corresponding $r(R_{M}^{mito})=0$. When a fusion event occurs between a given pair of mitochondria, one of the mitochondria is assigned randomly as the donor and the other as the acceptor mitochondrion. Subsequently, the nucleoids and subcompartments (if any) of the donor mitochondrion are appended to the acceptor, and a new fission site is created (see Fig 2*C*). Finally, the donor mitochondrion is removed from the corresponding cell compartment.

#### Fission

The fission propensity is calculated using the same function as in our previous study [42]. Under physiological conditions, mitochondria have been shown to contain between 1 and 10 nucleoids [69], and mitochondria with high nucleoid content are markedly less common than those with only a handful of nucleoids [70]. We therefore set the fission propensity of a mitochondrion to increase with its nucleoid content according to:

$$a_{fission,i}=V_{F,max}\frac{{\left(W_{i}+M_{i}\right)}^{n}}{K_{F}^{n}+{\left(W_{i}+M_{i}\right)}^{n}}\;.$$

where the parameter *V*_*F*,*max*_ is the maximum fission propensity. The sigmoidal dependence of fission on the nucleoid content is selected in order to reproduce a unimodal mitochondrial nucleoid distribution. In this study, the parameters *K*_*F*_ and *n* in the fission propensity above were determined according the procedure described in the next section.

In simulating fission, we follow our established model implementation [42]. For a previously fused mitochondrion, we select a fission site randomly (see Fig 2*C*) and exchange the nucleoid contents of two mitochondrial sub-compartments adjacent to the fission site based on a Binomial random number. The exchange of nucleoids is performed without any preference between WT and mutant mtDNA. Meanwhile, for mitochondria with no fission site, mtDNA are assigned to each daughter mitochondrion following a uniform random number, with a constraint that the daughter mitochondria should contain at least one nucleoid. Finally, one of the daughter mitochondria is placed in the original cellular compartment, while the other is randomly placed in either the same or in any of the neighboring cellular compartments.

While mitochondrial fusion-fission may not lead to any exchange of mtDNA nucleoids (referred to as kiss and run), this process still allows for the exchange of mitochondrial matrix components, such as proteins and solutes [71]. This fusion-assisted mixing can explain the complementation of mtDNA protein products among mitochondria, as mentioned above. In the present model, such mixing process is implicitly taken into account in the description for mitophagy selectivity. For example, a higher complementation among mitochondria that mitigates the impact of mtDNA mutations on OXPHOS, can be captured by increasing the parameter *K* in $s\left(R_{M}^{mito}\right)$. A higher *K* value implies that a higher fraction of mutant mtDNA is required to produce the same level of OXPHOS defects.

Furthermore, we did not explicitly assume any selectivity in the mitochondrial fission, i.e. in our model the fission propensity of a mitochondrion did not depend explicitly on the amount of mutant mtDNA. While the molecular mechanism of selectivity in mitochondrial fission is still unknown, a recent modeling cum experimental study has suggested that the fission rate of dysfunctional mitochondria is actually lower than that of functional mitochondria [51]. Despite the fact that our model did not contain an explicit mechanism or parameter implementing such dependence of fission rate on mutation burden, we found in our analysis of the model that mutation burden did indeed impact fission probability although through an indirect mechanism. This is because mutant-rich mitochondria with OXPHOS defects have low propensities to fuse and consequently, these mitochondria typically harbor a small number of nucleoids (see S2 Fig). By having fewer nucleoids, these mutant-rich mitochondria therefore have lower propensities to undergo fission than other (functional) mitochondria, thereby emulating selectivity of mitochondrial fission. In a future work, we might explore the above mechanism more fully by investigating mutation dependent differences in model parameters including fission propensity separately.

#### Fusion-fission parameters

We reproduced the process for assigning fusion-fission parameters as done in our previous study [42]. Briefly, the fusion-fission parameters were manually adjusted in two steps to reproduce experimentally observed distribution of mitochondrial sizes and mixing time of mitochondrial membrane protein. The parameter *V*_*F*,*max*_ of the fission propensity was set as a constant multiple of *a*_*fusion*,0_. By doing so, adjustments to parameter *a*_*fusion*,0_ would change how fast the mitochondrial population approaches steady state (mixing time), but would not affect the nucleoid distribution. Hence, the fusion parameter *a*_*fusion*,0_ could be specified separately from the fission parameters.

In the first step, we adjusted the parameters *K*_*F*_, *n* and *α* such that every mitochondrion contained between 1 to 10 nucleoids and the majority of mitochondria possessed 3–4 nucleoids. This is in accordance with the reported distribution of mitochondrial nucleoid content [72]. The size distribution of mitochondria at steady state resulting from the first step is shown in Fig 2*D*. Next the parameter *a*_*fusion*,0_ was tuned to reproduce experimentally observed time scale of mixing mitochondrial components. For example, differentially labeled mitochondrial membrane-bound proteins in fused HeLa cells take a few days to become well-mixed (see Fig 4 in [73]). In order to relate *a*_*fusion*,0_ with the rate of mixing of mtDNA, we carried out model simulations starting with half of the nucleoid population labelled as *W* and the other half as *M*. For these simulations, we have previously introduced a metric called *mixing time constant τ*, defined as the time for the coefficient of variation (COV) of R_M_^mito^, averaged over the population of cells, to reach 63.2% of its final steady state change [42]. In this case, a lower mixing time constant corresponds to a faster mixing rate due to more frequent fusion-fission events (i.e. a higher *a*_*fusion*,0_). The above simulations were performed for a range of values of *a*_*fusion*,0_ (see Fig 2*E*). The nominal value of *a*_*fusion*,0_ corresponding to a mixing time constant of 7.5 day is reported in Table 1.

#### Premixing simulations

In the premixing simulations, we initialized each cell with 32 mitochondria by placing two mitochondria in each cell compartment. We then assigned each mitochondrion with 10 nucleoids such that every cell simulation started with the same number of nucleoids (i.e. 320 nucleoids, see Table 1) [72, 74]. As the premixing simulations only involved mitochondrial fusion-fission, the total number of mtDNA would not vary. We simulated the premixing step for 50 days, which was the time taken for mitochondrial size distribution to reach steady state using the slowest fusion-fission rate in Table 2 (i.e. longest mixing time). The steady state distributions of mitochondria and nucleoids were not sensitive to the initial number of mitochondria and nucleoids per mitochondrion.

### Global parametric sensitivity analysis

In this study, we employed a variance-based global sensitivity analysis to map out the parametric dependence of the model output, which for the purpose of the study, we have set to be the average mutation burden ${\bar{R}}_{M}^{Cell}\left(t\right)$. Here, the sensitivities with respect to a parameter or a combination of parameters reflected the variance of ${\bar{R}}_{M}^{Cell}\left(t\right)$ that was attributed to the (co-)variability in the respective parameter(s). More specifically, we calculated the following first and second order sensitivity coefficients [75]:

$$S_{i}\left(t\right)=\frac{V\left[E\left({\bar{R}}_{M}^{Cell}\;\left(t\right)|p_{i}\right)\right]}{V\left({\bar{R}}_{M}^{Cell}\;\left(t\right)\right)}$$

$$S_{ij}\left(t\right)=\frac{V\left[E\left({\bar{R}}_{M}^{Cell}\;\left(t\right)|p_{i},p_{j}\right)\right]-V\left[E\left({\bar{R}}_{M}^{Cell}\;\left(t\right)|p_{i}\right)\right]-V\left[E\left({\bar{R}}_{M}^{Cell}\;\left(t\right)|p_{j}\right)\right]}{V\left({\bar{R}}_{M}^{Cell}\;\left(t\right)\right)}$$

where *E*(⋅) and *V*(⋅) are the expectation and variance functions, respectively. Here, *S*_*i*_ (*t*) is the first order sensitivity coefficient of ${\bar{R}}_{M}^{Cell}\left(t\right)$ with respect to the parameter *p*_*i*_, and *S*_*ij*_ (*t*) is the second order sensitivity coefficient of ${\bar{R}}_{M}^{Cell}\left(t\right)$ with respect to the parameters *p*_*i*_ and *p*_*j*_. In the GSA of the mitochondrial QC model, we employed a Latin hypercube sampling to generate 2048 distinct parameter combinations from the parameter ranges given in Table 2. The parameters *K* and *m* associated with the mitophagy selectivity, fusion selectivity and retrograde signaling function, i.e. *K*_*D*_, *K*_*fusion*_ and *K*_*R*_, respectively, were perturbed equally to ensure the same OXPHOS defect threshold in the propensities. We performed model simulations for each parameter combination, generating 2048 trajectories of ${\bar{R}}_{M}^{Cell}\left(t\right)$. Finally, we computed *S*_*i*_ (*t*) and *S*_*ij*_ (*t*) using the GUI-HDMR toolbox in MATLAB [75].

## Supporting Information

S1 Fig Comparison of model simulations using circular and cylindrical cell geometries. (PDF)

S2 Fig Comparison of mitochondrial nucleoid content between mutant-rich mitochondria (with mutant fractions >90%) and the rest of the mitochondrial population in model simulations with *τ* = 7.5 and 30 days. (PDF)

S1 Table Sensitivity coefficients of global parametric sensitivity analysis. (PDF)
