# Supplementary figures and images for: Context-Dependent Role of Mitochondrial Fusion-Fission in Clonal Expansion of mtDNA Mutations
Source: PLoS Comput Biol. 2015 May 21;11(5):e1004183. doi: 10.1371/journal.pcbi.1004183 (PMC4440705; doi:10.1371/journal.pcbi.1004183)

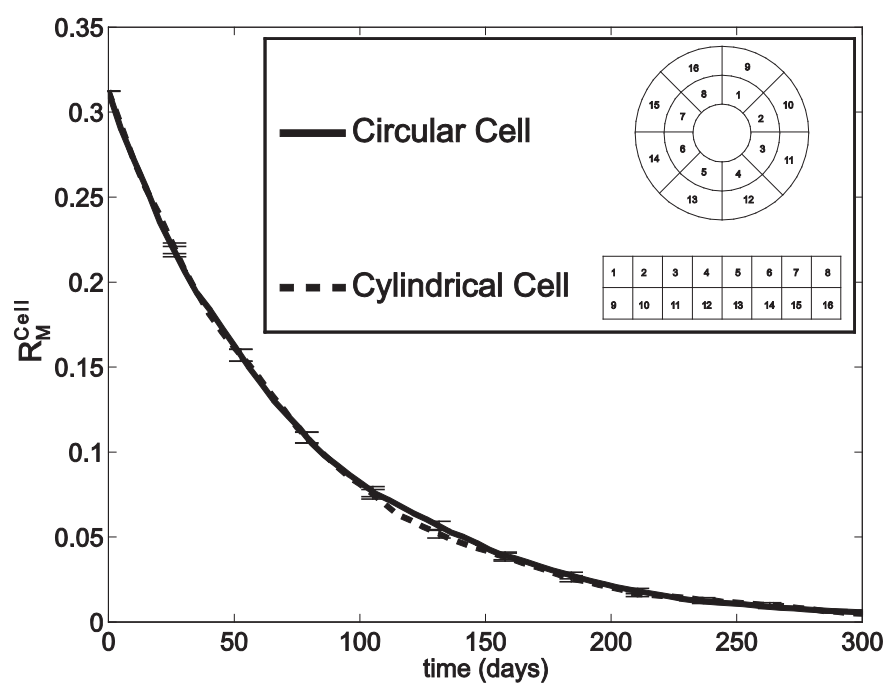

Figure S1. Comparison of model simulations using circular and cylindrical cell geometries.

Supplement: S1 Fig — (PDF) [file pcbi.1004183.s001.pdf]
