# Supplementary material for: Context-Dependent Role of Mitochondrial Fusion-Fission in Clonal Expansion of mtDNA Mutations
Source: PLoS Comput Biol. 2015 May 21;11(5):e1004183. doi: 10.1371/journal.pcbi.1004183 (PMC4440705; doi:10.1371/journal.pcbi.1004183)
Supplement: S2 Fig — (PDF) [file pcbi.1004183.s002.pdf]

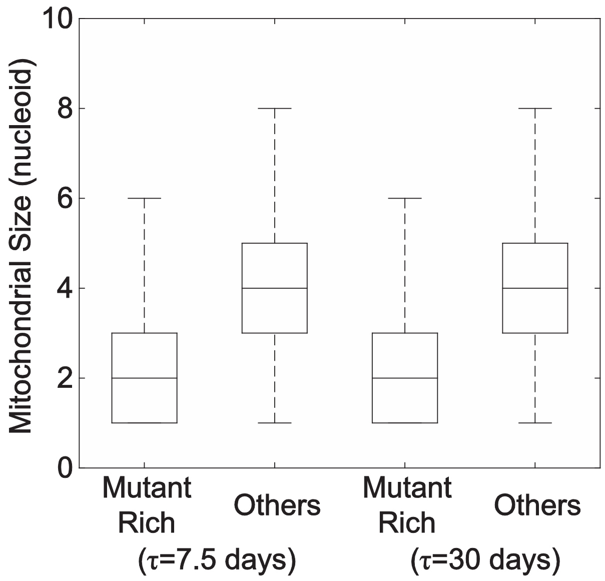

Figure S2. Comparison of mitochondrial nucleoid content between mutant-rich mitochondria (with mutant fractions >90%) and the rest of the mitochondrial population in model simulations with $\tau = 7.5$ and 30 days.
