# Supplementary material for: Context-Dependent Role of Mitochondrial Fusion-Fission in Clonal Expansion of mtDNA Mutations
Source: PLoS Comput Biol. 2015 May 21;11(5):e1004183. doi: 10.1371/journal.pcbi.1004183 (PMC4440705; doi:10.1371/journal.pcbi.1004183)
Supplement: S1 Table — (PDF) [file pcbi.1004183.s003.pdf]

Supporting Table

Context-Dependent Role of Mitochondrial Fusion-Fission in Clonal Expansion of mtDNA Mutations

Zhi Yang Tam, Jan Gruber, Barry Halliwell, Rudiyanto Gunawan

Table S1. Sensitivity Coefficients of Global Parametric Sensitivity Analysis

Parameters	Day 50	Day 100	Day 150	Day 200	Day 250
**Replicative advantage (k_R)**	4.32x10^{-1}	4.15x10^{-1}	3.99x10^{-1}	3.80x10^{-1}	3.59x10^{-1}
**Mitophagy selectivity strength ($r_{D,max}$)**	2.33x10^{-1}	2.27x10^{-1}	2.23x10^{-1}	2.18x10^{-1}	2.12x10^{-1}
**Fusion selectivity strength ($r_{fusion,max}$)**	1.24x10^{-1}	1.27x10^{-1}	1.22x10^{-1}	1.16x10^{-1}	1.08x10^{-1}
**Selectivity threshold (K_D) (Mitophagy)**	7.03x10^{-2}	7.45x10^{-2}	7.32x10^{-2}	7.13x10^{-2}	6.91x10^{-2}
**Replicative advantage (k_R) + Mitophagy selectivity strength ($r_{D,max}$)**	0	1.24x10^{-2}	2.06x10^{-2}	3.06x10^{-2}	4.35x10^{-2}
**Mixing time constant (τ)**	2.19x10^{-2}	3.16x10^{-2}	3.56x10^{-2}	3.82x10^{-2}	3.93x10^{-2}
**Replicative advantage (k_R) + Mixing time constant (τ)**	0	1.08x10^{-2}	1.2x10^{-2}	1.39x10^{-2}	1.59x10^{-2}
**Replicative advantage (k_R) + Fusion selectivity strength ($r_{fusion,max}$)**	0	0	2.93x10^{-3}	9.07x10^{-3}	1.54x10^{-2}
**Mitophagy selectivity strength ($r_{D,max}$) + Selectivity threshold (K_D) (Retrograde signaling)**	0	0	0	0	2.05x10^{-2}
**Replicative advantage (k_R) + Mitophagy rate constant (k_D)**	4.37x10^{-2}	2.79x10^{-2}	2.17x10^{-2}	1.8x10^{-2}	1.51x10^{-2}
**Fusion selectivity strength ($r_{fusion,max}$) + Retrograde signaling strength ($r_{R,max}$)**	0	0	0	0	6.34x10^{-3}
**Replicative advantage (k_R) + Selectivity threshold (K_D) (Mitophagy)**	0	0	1.3x10^{-3}	4.7x10^{-3}	8.55x10^{-3}
**Mitophagy selectivity strength ($r_{D,max}$) + Fusion selectivity strength ($r_{fusion,max}$)**	0	0	0	0	0
**Mitophagy rate constant (k_D) + Selectivity threshold (K_D) (Mitophagy)**	4.01x10^{-2}	2.21x10^{-2}	1.64x10^{-2}	1.37x10^{-2}	1.2x10^{-2}
**Mitophagy rate constant (k_D) + Fusion selectivity strength ($r_{fusion,max}$)**	0	0	2.07x10^{-2}	1.55x10^{-2}	1.25x10^{-2}
**Selectivity threshold (K_D) (Fusion)**	9.71x10^{-3}	1.09x10^{-2}	1.1x10^{-2}	1.07x10^{-2}	1.03x10^{-2}
**Mixing time constant (τ) + Mitophagy selectivity strength ($r_{D,max}$)**	0	9.43x10^{-5}	1.04x10^{-3}	2.47x10^{-3}	6.07x10^{-3}
**Mitophagy selectivity threshold (K_D) + Selectivity threshold (K_D) (Fusion)**	0	0	0	1.06x10^{-2}	8.49x10^{-3}
**Mitophagy rate constant (k_D) + Mitophagy selectivity strength ($r_{D,max}$)**	4.43x10^{-3}	6.51x10^{-3}	7.21x10^{-3}	7.35x10^{-3}	7.34x10^{-3}
**Selectivity threshold (K_D) (Retrograde signaling)**	0	4.45x10^{-3}	4.46x10^{-3}	5.7x10^{-3}	5.98x10^{-3}
**Mixing time constant (τ) + Selectivity threshold (K_D) (Fusion)**	0	1.88x10^{-2}	1.16x10^{-2}	8.5x10^{-3}	6.78x10^{-3}
**Mixing time constant (τ) + Selectivity threshold (K_D) (Mitophagy)**	0	2.91x10^{-3}	2.49x10^{-3}	2.93x10^{-3}	3.52x10^{-3}
**Mixing time constant (τ) + Retrograde signaling strength ($r_{R,max}$)**	0	0	0	0	5.18x10^{-3}
